# Robust transformation procedure for the production of transgenic farmer-preferred cassava landraces

**DOI:** 10.1186/1746-4811-8-24

**Published:** 2012-07-11

**Authors:** Ima M Zainuddin, Kim Schlegel, Wilhelm Gruissem, Hervé Vanderschuren

**Affiliations:** 1 Department of Biology, Plant Biotechnology, ETH Zurich-LFW E56.1, Universitaetstrasse 2, 8092, Zurich, Switzerland

**Keywords:** Cassava, Tropical crop, Genetic transformation, Somatic embryogenesis, Agrobacterium, Farmer-preferred landraces

## Abstract

Recent progress in cassava transformation has allowed the robust production of transgenic cassava even under suboptimal plant tissue culture conditions. The transformation protocol has so far been used mostly for the cassava model cultivar 60444 because of its good regeneration capacity of embryogenic tissues. However, for deployment and adoption of transgenic cassava in the field it is important to develop robust transformation methods for farmer- and industry-preferred landraces and cultivars. Because dynamics of multiplication and regeneration of embryogenic tissues differ between cassava genotypes, it was necessary to adapt the efficient cv. 60444 transformation protocol to genotypes that are more recalcitrant to transformation. Here we demonstrate that an improved cassava transformation protocol for cv. 60444 could be successfully modified for production of transgenic farmer-preferred cassava landraces. The modified transformation method reports on procedures for optimization and is likely transferable to other cassava genotypes reportedly recalcitrant to transformation provided production of high quality FEC. Because the three farmer-preferred cassava landraces selected in this study have been identified as resistant or tolerant to cassava mosaic disease (CMD), the adapted protocol will be essential to mobilize improved traits into cassava genotypes suitable for regions where CMD limits production.

## Introduction

Cassava is the staple food for nearly a billion people in 105 countries [1]. Because of its resilience and capacity to grow on marginal lands, the importance of cassava cultivation in farming systems affected by climate change is expected to increase in the future [2]. The use of cassava as energy crop also contributes to its increasing production acreage in tropical countries [3,4]. However, the lack of resistance genes in the available germplasm, high heterozygosity, allopolyploidy, low fertility, and unsynchronized flowering make cassava improvement by conventional breeding a long and tedious process [5,6]. Therefore cassava genetic transformation has emerged as a valuable alternative and complementary approach to improve cassava [7,8]. Several protocols using either cotyledons or embryogenic cultures as target tissues and particle bombardment or *Agrobacterium*-mediated transformation procedures have been reported in the literature [8]. However the use of embryogenic tissues (i.e. friable embryogenic callus, FEC) in combination with *Agrobacterium*-mediated transformation has become the favoured method because of its higher efficiency compared to the cotyledon-based protocol [9-13]. Despite this progress cassava remains difficult to transform partly as the result of low transformation and regeneration frequencies. The *Agrobacterium*-FEC system is also unstable and in some conditions produces highly variable numbers of transgenic events [12]. As a consequence, cassava transformation requires well-trained tissue culture specialists, substantial amounts of plant material and repeated transformation cycles to generate a sufficient number of independent transgenic lines for research and product development. The instability of the transformation system renders the establishment of cassava transformation technology under less favourable conditions more challenging [14-16]. Recent progress in the optimization of the transformation protocol has substantially increased efficiency and robustness [17,18]. The improved transformation protocol was subsequently established in laboratories located in Africa based on hands-on workshops and training of local scientists [15,16].

The majority of transgenic cassava reports have been based on the transformation of the model cultivar 60444 (previously referred to as TMS 60444) [8]. While proof-of-concept is possible with the model cultivar, the importance of transforming farmer- and industry-preferred cassava cultivars is essential for the adoption of transgenic cassava [7,15,16]. Because transgenic strategies to improve cassava are now being evaluated in the field [9,12,13] it is also important to assess the technology in cassava genotypes adapted to the respective field environments. Locally adapted cultivars and landraces have often been selected and adopted by farmers because of particular improved traits [19]. Production of transgenic events in those selected genotypes offer the possibility to rapidly stack improved traits. Improvement of farmer-preferred genotypes using *Agrobacterium*-mediated transformation of FEC, however, has been limited by the difficulty of generating plant tissue suitable for transformation, the low regeneration efficiency of FEC, and the time necessary for embryo maturation following their co-cultivation with *Agrobacterium*[8]. In particular, FEC initiation and time to regeneration are genotype dependent [20,21].

Here we describe a modified and efficient method for transformation of three farmer-preferred cassava landraces that were selected based on their virus resistance [19,22] as well as preferential and extensive use in Africa. Production of tissues suitable for transformation were generated and tested for regeneration. Because their multiplication and regeneration dynamics differed from cv. 60444, a modified transformation protocol was developed.

## Materials

### Reagents

#### Plant material

Shoot cultures of cv. 60444 and three farmer-preferred cassava landraces (2^nd^Agric (TME3), Oko-iyawo (TME7), and Abbey-ife (TME14)) [19] were obtained from the ETH Zurich and International Institute of Tropical Agriculture (IITA, Nigeria) *in vitro* cassava germplasm collections.

#### Bacteria

*Agrobacterium tumefaciens* LBA4404 harboring CAMBIA 1301 plasmid (GeneBank AF234297) which contains the *hptII* gene for resistance to hygromycin and the *gusA* reporter gene driven by the constitutive 35 S promoter.

#### Media

· CBM (basic shoot culture medium), for propagation of in vitro plantlets: 1× MS salts with vitamins, 2 μM CuSO_4_, 2% sucrose, 0.3% Gelrite, pH 5.8, autoclaved

· CAM (axillary bud enlargement medium), for induction of axillary buds: 1× MS salts with vitamins, 2 μM CuSO_4_, 10 mg/l BAP, 2% sucrose, 0.8% Noble agar, pH 5.8, autoclaved

· CEM (shoot elongation medium) for shoot elongation: 1x MS salts with vitamins, 2 μM CuSO_4_, 0.4 mg/l BAP, 2% sucrose, 0.8% Noble agar, pH 5.8, autoclaved

· CIM (somatic embryo induction medium) for induction of somatic embryos: 1× MS salts with vitamins, 2 uM CuSO_4_, 12 mg/l picloram, 2% sucrose, 0.8% Noble agar, pH 5.8, autoclaved

· GD (friable embryogenic callus medium) for induction and propagation of FEC: 1x GD salts with vitamins, 12 mg/l picloram, 2% sucrose, 0.8% Noble agar, pH 5.8, autoclaved

· GD (friable embryogenic callus medium) liquid medium for dilution of bacteria and FEC washing steps: 1× GD salts with vitamins, 12 mg/l picloram, 2% sucrose, pH 5.8, autoclaved

· MSN (somatic embryo emerging medium) for regeneration of embryos: 1× MS salts with vitamins, 1 mg/l NAA, 2% sucrose, 0.8% Noble agar, pH 5.8, autoclaved

· YEB (yeast extract broth) solid medium for *Agrobacterium* selection: 1 g/l Bacto^TM^ yeast extract, 5 g/l Bacto^TM^ beef extract, 5 g/l Bacto^TM^ peptone, 5 g/l sucrose, 1.5% Bacto^TM^ agar, pH 7.2, autoclaved

· YEB (yeast extract broth) liquid medium for Agrobacterium culture: 1 g/l Bacto^TM^ yeast extract, 5 g/l Bacto^TM^ beef extract, 5 g/l Bacto^TM^ peptone, 5 g/l sucrose, pH 7.2, autoclaved. Supplemented with MgSO_4_ sterile solution (2 mM final concentration)

#### Other chemicals

· Antibiotics for bacterial selection: kanamycin 50 mg/mL solution, rifampicin 25 mg/mL solution, and streptomycin 100 mg/mL solution

· Antibiotics for prevention of bacterial growth: carbenicillin 500 mg/mL solution.

· Antibiotics for selection of transformed cassava FEC and rooting test of transgenic cassava plantlets: hygromycin 15 mg/mL solution.

· Acetosyringone 200 mM solution

· GUS reaction buffer: 10 mM Tris (pH7.2), 50 mM NaCl, 0.1% Triton X-100, and 0.5 mg/mL 5-bromo-4-chloro-3-indolyl glucuronide

· Reagents for Southern blot: *Hind*III and *Pst*I restriction enzymes, 1% agarose gel, Hybond-N+ membrane (GE Healthcare), DNA probe (*hptII* DNA probe labeled with the DIG labelling mix (Roche))

#### Plasticware & other consumables

· Sterile plastic Petri dishes, 90 mm

· Plastic mesh 100 μm, sterile

· Pipettes 25 mL, sterile

· Sterile jars

· 15 mL and 50 mL sterile disposable tubes

· Eppendorf tubes (1.5 mL)

· Parafilm

· Sterile disposable syringe filters (0.22 μm)

· Aluminium foil

· Disposable syringes (10 mL)

· Sterile, disposable 1 mL tips

· Films (Kodak BioMax Light Film)

#### Equipments

· Autoclave

· pH meter

· Precision balance

· Centrifuge for 50 mL tubes

· Shaker

· Controlled environment chamber/room (28°C, 16 h light/8 h dark)

· Controlled environment chamber/room (24°C, 16 h light/8 h dark)

· Pipette aid

· Laminar flow hood with Bunsen burner

· Fridge (4°C) and freezer (−20°C)

· Spatula, scalpel, and forceps

· Binocular microscope

· Inoculation loops

· Incubator-shaker (28°C)

· Spectrophotometer

· 1 mL disposable cuvettes

· Magnetic stirring bars

· Vortexer

· Micropipette

· PCR machine

· UV – crosslinker (BIO-LINK^™^)

· Processor (Agfa Curix 60)

### Protocols

#### Production of somatic embryos from cv. 60444, 2^nd^Agric, Oko-iyawo, and Abbey-ife

**Figure 1:**
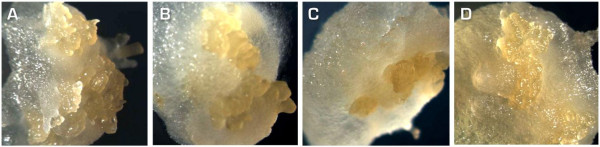
**Primary somatic embryos of cassava genotypes: A. cv. 60444, B. 2**^**nd**^**Agric, C. Oko-iyawo, D. Abbey-ife.**

1. Take 5-10 mm long stem cuttings from 4-week-old *in vitro* plantlets of selected cassava cultivars and place them horizontally on Petri dishes containing CAM for 2-4 days at 28°C in the dark.

2. Remove the enlarged axillary buds from the nodal explants with sterile syringe needles under a binocular microscope and transfer them to Petri dishes containing CIM. Keep them for two weeks at 28°C in the dark.

3. Subculture the developing embryos (Figure 1) with sterile syringe needles. Use a binocular microscope to remove callus developing around the embryos. Place the embryos on fresh CIM at 28°C in the dark. Do this step at two-week intervals for multiplication of the embryos and initiation of cyclic secondary somatic embryogenesis.

**NOTE:** Cyclic embryogenesis is a routine method of *de novo* plant regeneration *in vitro* and is particularly adequate to provide a constant source of pure embryo clusters necessary for the induction of friable embryogenic callus (FEC). Four cycles on CIM usually generate sufficient quantities of pure secondary somatic embryos required for FEC induction of all selected landraces.

#### Production and proliferation of FEC

This step is a key parameter of the protocol because FEC production as well as their capacity to proliferate and regenerate are strongly genotype-dependent [16,20,23].

1. Transfer an equal amount of secondary embryos from each genotype to GD medium and incubate them at 28°C in the dark. Check the plates visually under a binocular microscope to identify the developing FEC. Do this step every week (up to four weeks) after transfer to GD medium since FEC initiation and development is genotype-dependent. **OUR RESULTS:** Table 1 shows the percentage of FEC initiated on GD medium for the selected cassava landraces. Even though high quality secondary somatic embryos were used for all farmer-preferred landraces, their potential for FEC generation was significantly lower when compared to cv. 60444.

**Table 1:** FEC induction results of the selected cassava genotypes

**Name**	**Somatic Embryos (clusters)**	**FEC (clumps)**	**Percentage (%)**
cv. 60444	168	128	76
2nd Agric	244	24	11
Oko-iyawo	168	32	19
Abbey-ife	232	24	10

Each plate contains 9 clusters of somatic embryo or 9 clumps of FEC.

**Figure 2:**
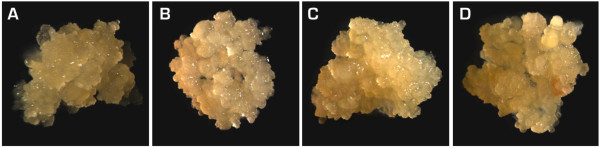
**Proliferating FEC from cassava genotypes: (A) cv. 60444, (B) 2**^**nd**^**Agric, (C) Oko-iyawo, (D) Abbey-ife**.

**Figure 3:**
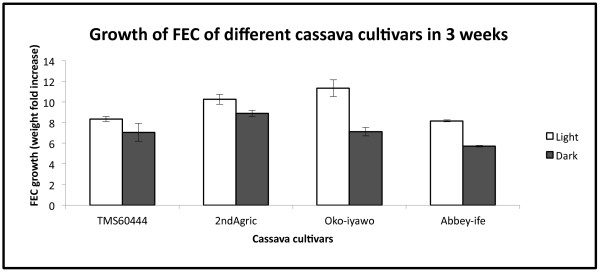
**FEC growth after three weeks on GD medium.** Each value represents the mean ± SE of two plates, each with 4 FEC clumps.

**Figure 4:**
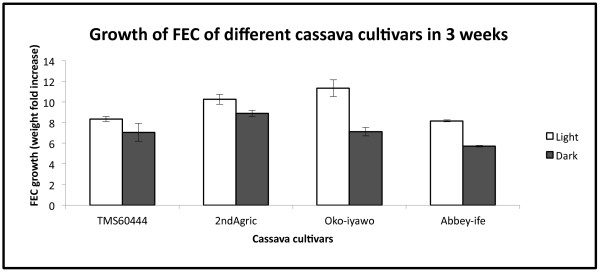
**Number of emerging embryos from cassava FEC clumps.** Each value is the mean ± SE of two plates, each with 4 clumps of FEC.

2. Subculture the embryos to fresh GD at three weeks interval to purify and multiply high quality FEC. Multiply the FEC over 2-3 cycles on GD medium in the dark at 28°C to obtain high quality and pure FEC (Figure 2).

3. To determine the optimal light regime, incubate the high quality FEC in 16 h photoperiod or in the dark (see next steps).

4. Measure their weight increase after 2-3 weeks which corresponds to the typical proliferation time for multiplication of cv. 60444 FEC. OUR RESULTS: In the light FEC from 2^nd^Agric and Oko-iyawo landraces showed a higher weight increase than FEC from cv. 60444 (Figure 3). While production of FEC differed substantially between cv. 60444 and the farmer-preferred landraces (Table 1), selection of high quality FEC could overcome those limitations by rapid proliferation on GD medium. The proliferation of FEC in the dark was slower than in the light.

5. Transfer FEC to a low auxin medium for maturation (i.e. somatic embryo emerging medium, MSN).

6. Count the number of emerging embryo per FEC clump every week (up to 5-6 weeks).

7. Compare the proliferation and regeneration rate of the selected landraces between the light and dark regime to determine the influence of light regime on FEC regeneration capacity. OUR RESULTS: The dark regime for FEC multiplication did have a negative effect on cv. 60444 embryo maturation potential. On the contrary 2^nd^Agric FEC regenerated a significantly higher number of maturing embryos when initially proliferated in the dark (Figure 4). Because of the positive or neutral effect of FEC proliferation in the dark on the embryo regeneration rate for the selected farmer-preferred landraces (Figure 4), all selective steps on FEC proliferation media were performed in the dark.

### *Agrobacterium*-mediated transformation

#### Preparation of *Agrobacterium* culture for inoculation

1. Inoculate a single colony of *A. Tumefaciens* LBA4404 harboring pCAMBIA1301 into 5 mL of YEB liquid medium containing the following antibiotics: kanamycin 50 mg/L, rifampicin 50 mg/L and streptomycin 100 mg/L. Keep the culture at 28°C with 200 rpm agitation overnight.

2. Take 1 mL of the overnight culture to inoculate 25 mL of YEB liquid medium supplemented with the aforementioned antibiotics in 250 mL flask under the same growth conditions.

3. After reaching an OD_600_ of 0.7-1.0, pellet the bacteria by centrifugation in 50 mL tubes at 5000 rpm for 10 min.

4. Remove the supernatant and resuspend the bacteria pellet in GD liquid medium to remove antibiotics. Centrifuge the bacteria suspension at 5000 rpm for 10 min.

5. Remove the supernatant and resuspend the bacteria pellet in GD liquid medium to a final OD_600_ of 0.5.

6. Add acetosyringone to the *Agrobacterium* culture to a final concentration of 200 μM.

7. Shake the cultures at 50 rpm for 45 min at room temperature.

#### *Agrobacterium* inoculation and co-cultivation of FEC

1. Drop the bacteria suspension with 1 mL pipette on FEC clumps until each clump becomes wet and remove the excess of bacteria suspension. Seal the Petri dishes and incubate at 22-23°C for 3 days under the optimal light/dark regime (16 h photoperiod for the cv. 60444 FEC and in the dark for the farmer-preferred landraces).

2. Scrape the inoculated FEC using sterile forceps and place them in 25 mL GD liquid medium containing carbenicillin 500 mg/L. Use sterile 50 mL tubes for this step.

3. Gently vortex the suspension for 5-10 s and then allow the FEC to pellet.

4. Remove the supernatant GD medium and repeat the washing step until the GD medium becomes clear and transparent. It usually requires 4-5 washing steps.

5. Resuspend the cleaned FEC in GD solution with a 25 mL sterile pipet and spread them evenly on a sterile 100 μm nylon mesh.

6. Transfer the mesh with the FEC layer on sterile filter paper to remove excess GD medium.

7. Place the mesh with FEC on GD plate containing carbenicillin 250 mg/L for 3-4 days at 28°C under optimal light/dark regime (16 h photoperiod for the cv. 60444 FEC and in the dark for the farmer-preferred landraces).

#### Gradual selection of transformed FEC

By comparing the performance of two different treatments, single-step (SS) gradual selection, which corresponds to the procedure previously described by Bull and colleagues [17], and double-step (DS) gradual selection in which the time on gradual selection was doubled, we showed that doubling the time of recovery and proliferation substantially increased the number of maturing embryos on MSN medium in the following regeneration steps (Table 2). Hence we outlined below the procedure of DS gradual selection for the selected cassava landraces:

1. Transfer the mesh with FEC to GD plates supplemented with carbenicillin 250 mg/L and hygromycin 5 mg/L. Place them in the dark at 28°C for 2 weeks with medium replenishment every week.

2. Transfer the mesh with FEC to GD plates supplemented with carbenicillin 250 mg/L and hygromycin 8 mg/L. Place them in the dark at 28°C for 2 weeks with medium replenishment every week.

3. Transfer the mesh with FEC to GD plates supplemented with carbenicillin 250 mg/L and hygromycin 15 mg/L. Place them in the dark at 28°C for 2 weeks with medium replenishment every week.

**Table 2:** **Assessment of single-step (SS) and double-step (DS) selection procedure using the*Agrobacterium*-FEC transformation procedure with the selected genotypes**

**Genotype**	**Procedure**	**Cumulative number**	**Amount of FEC of maturing embryos (clumps)**
cv. 60444	SS	18	58
cv. 60444	DS	18	122
2nd Agric	SS	18	8
2nd Agric	DS	18	32
Oko-iyawo	SS	18	23
Oko-iyawo	DS	18	57

#### Selection and regeneration of transformed plantlets

1. Transfer the mesh with FEC to MSN plates supplemented with carbenicillin 250 mg/L and hygromycin 15 mg/L. Incubate at 28°C in 16 h photoperiod for 7-10 days.

2. Repeat step 1 up to six times. Small, greenish tube-like structures will start appearing after 2-4 cycles on MSN. Retain them on the mesh until development of green cotyledons.

3. Use sterile forceps or syringe needles under the binocular microscope to transfer the matured embryos with expanded green cotyledons to CEM solid medium supplemented with carbenicillin 100 mg/L.

#### Histochemical GUS assay

To determine the percentage of transgenic maturing embryos appearing on the regeneration medium, embryos can be selected and tested using the GUS assay by following the steps as earlier described [18]:

1. Immerse the randomly selected matured embryos in GUS reaction buffer.

2. Incubate at 37°C overnight.

3. Remove the GUS buffer and wash the embryos with 70% ethanol solution.

OUR RESULTS: The percentage of GUS positive embryos varied between the selected landraces (Table 3). For all selected landraces more than half of the embryos were GUS positive.

**Table 3:** GUS assay, rooting test, and independent line percentage of the selected cassava landraces

**Name**	**Procedure**	**Number of tested maturing embryo**	**GUS Positive**	**Number of regenerated shoots**	**Rooting Test positive**	**Transformation efficiency**		
						**GUS assay**	**Rooting Test**	**Independent Line**
2nd Agric	DS	17	9	22	17	53%	77%	63%
Oko-iyawo	DS	20	16	18	13	80%	72%	50%
Abbey-ife	DS	20	14	9	7	70%	78%	100%
Oko-iyawo	SS	16	14	10	9	87%	90%	67%

Single-step (SS) and double-step (DS) on proliferation media are reported in the procedure column.

## Rooting test

This procedure is an easy method for rapid and reliable screening of transgenic cassava lines [17,24]:

1. Cut the shoots elongating on CEM and transfer them to CBM jars supplemented with carbenicillin 50 mg/L and hygromycin 10 mg/L. Use the plantlets of non transformed wild-type cassava as negative control.

2. Maintain the plantlets under 16 h photoperiod at 28°C.

3. Check the appearance of adventitious roots on cuttings at 2 weeks after planting. Transgenic shoots develop adventitious roots while non transgenic shoots turn brownish at the stem cut.

**Figure 5:**
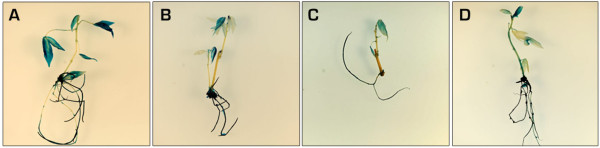
**GUS staining of transformed cassava genotypes: (A) cv. 60444, (B) 2**^**nd**^**Agric, (C) Oko-iyawo, (D) Abbey-ife.**

OUR RESULTS: Over 70% of the regenerated shoots from all selected landraces were positive for the rooting test (Table 3). GUS assay was also performed to confirm the transgenic status of regenerated plantlets positive in the rooting test (Figure 5).

## Southern blot analysis

Southern Blot analysis is performed to determine the number of independent insertion events for each plantlet positive in the rooting test. This molecular analysis is carried out following previously described methods [25,26]:

1. Extract the cassava genomic DNA from freeze-dried leaves.

2. Digest 10 μg DNA with 25000 units of *Hind*III or 20000 units *Pst*I overnight and run the digested DNA in 1% agarose gel.

3. Blot the DNA onto Hybond-N+ membrane (GE Healthcare) and fix by cross-linking in a BIO-LINK^™^ UV-crosslinker.

4. Hybridize the membrane with DIG-labeled probe amplified from the hygromycin gene present in pCAMBIA1301.

5. Reveal presence of hybridized probes with light sensitive films.

## Trouble shooting

Doubling the recovery and proliferation time on selective medium increases the probability of regenerating transgenic plantlets from the same transgenic event. In our hands, Oko-iyawo produced a lower percentage of independent transformation events (Table 3 & Additional file 1: Figure S1). It was noticeable that the extended time on proliferation media resulted in a thick layer of FEC and therefore FEC were most probably not homogenously exposed to antibiotic selection. Thick FEC layers can increase escape rates [16] and therefore it is recommended to spread the FEC layer (*Agrobacterium* inoculation and co-cultivation of FEC, step 5) as thin as possible (1-2 mm thick).

The optimization of the cassava transformation protocol for farmer-preferred landraces was performed with 18 FEC clumps, which represents a limited amount of starting material. For applications requiring a large number of independent transgenic plantlets, the amount of high quality FEC clumps can be scaled up in order to produce the required number of independent transgenic lines. This is particularly important for field experiment research and product development, for which precise transgene expression levels and single integration events are needed. We routinely used between 40 and 100 FEC clumps as starting material to generate over 20 independent transgenic lines per construct. For example, we recently used the protocol described here to generate transgenic cassava Oko-iyawo lines resistant to cassava brown streak disease (CBSD) using 45 FEC clumps to generate over 20 independent transgenic lines [27].

## Comments

The optimized transformation procedure for cv. 60444 [17] allowed us to establish a high-throughput transformation platform at ETH Zurich as well as transfer of this technology to African laboratories [15,16]. The development of a genotype-independent transformation procedure has been recognized by the cassava research community as an essential step for the adoption and deployment of transgenic cassava lines with improved traits [7,8,15]. Because of its robustness and efficiency, the improved transformation protocol will also be adequate for the production of transgenic events in other farmer-preferred cultivars and landraces. Adapting the protocol to maintain a high efficiency at each key step of the *Agrobacterium*-FEC based transformation, we demonstrated here that our method is also suitable for the production of transgenic farmer-preferred landraces. The efficiency and reliability of the adapted protocol for the production of independent transgenic events in farmer-preferred landraces is comparable to cv. 60444 and therefore is transferable to other laboratories as well. A robust transformation protocol for farmer-preferred cassava cultivars and landraces will be particularly beneficial for laboratories located in regions where cassava is used as food security and energy crop.

The optimization procedure and the protocol described here can also be used to establish the transformation technology for additional farmer- and industry-preferred cultivars in other laboratories. Our recent technology transfer activities in South Africa and Kenya demonstrated already that the protocol is robust and suitable for the production of transgenic lines from a locally grown industry-preferred cultivar [16]. Because the landraces presented in the study were selected on the basis of their CMD resistance trait, our method will be instrumental for stacking genetic and engineered traits, for which proof-of-concept already exists.

## Competing interests

The authors declare that they have no competing interests.

## Authors’ contributions

HV and IMZ designed the experiments; IMZ and KS undertook experimental works; HV and IMZ wrote the manuscript; WG edited the manuscript. All authors read and approved the final manuscript.

## Supplementary Material

Additional file 1 **Figure S1.** Southern blot analysis of transgenic cassava plantlets. Molecular analysis of transgenic plantlets from (A) 2^nd^Agric genotype using the double-step procedure, (B) Oko-iyawo genotype using the double-step procedure, (C) Abbey-ife genotype using the double-step procedure, (D) Oko-iyawo genotype using the single-step procedure. Independent lines are indicated by red labels. Southern blot has been repeated with *Pst*I restriction for lines with similar integration pattern to confirm independent integration event (data not shown). (PDF 9651 kb) Click here for file
