# Supplementary material for: Robust transformation procedure for the production of transgenic farmer-preferred cassava landraces
Source: Plant Methods. 2012 Jul 11;8:24. doi: 10.1186/1746-4811-8-24 (PMC3439245; doi:10.1186/1746-4811-8-24)
Supplement: Additional file 1 — Figure S1. Southern blot analysis of transgenic cassava plantlets. Molecular analysis of transgenic plantlets from (A) 2ndAgric genotype using the double-step procedure, (B) Oko-iyawo genotype using the double-step procedure, (C) Abbey-ife genotype using the double-step procedure, (D) Oko-iyawo genotype using the single-step procedure. Independent lines are indicated by red labels. Southern blot has been repeated with PstI restriction for lines with similar integration pattern to confirm independent integration event (data not shown). (PDF 9651 kb) [file 1746-4811-8-24-S1.pdf]

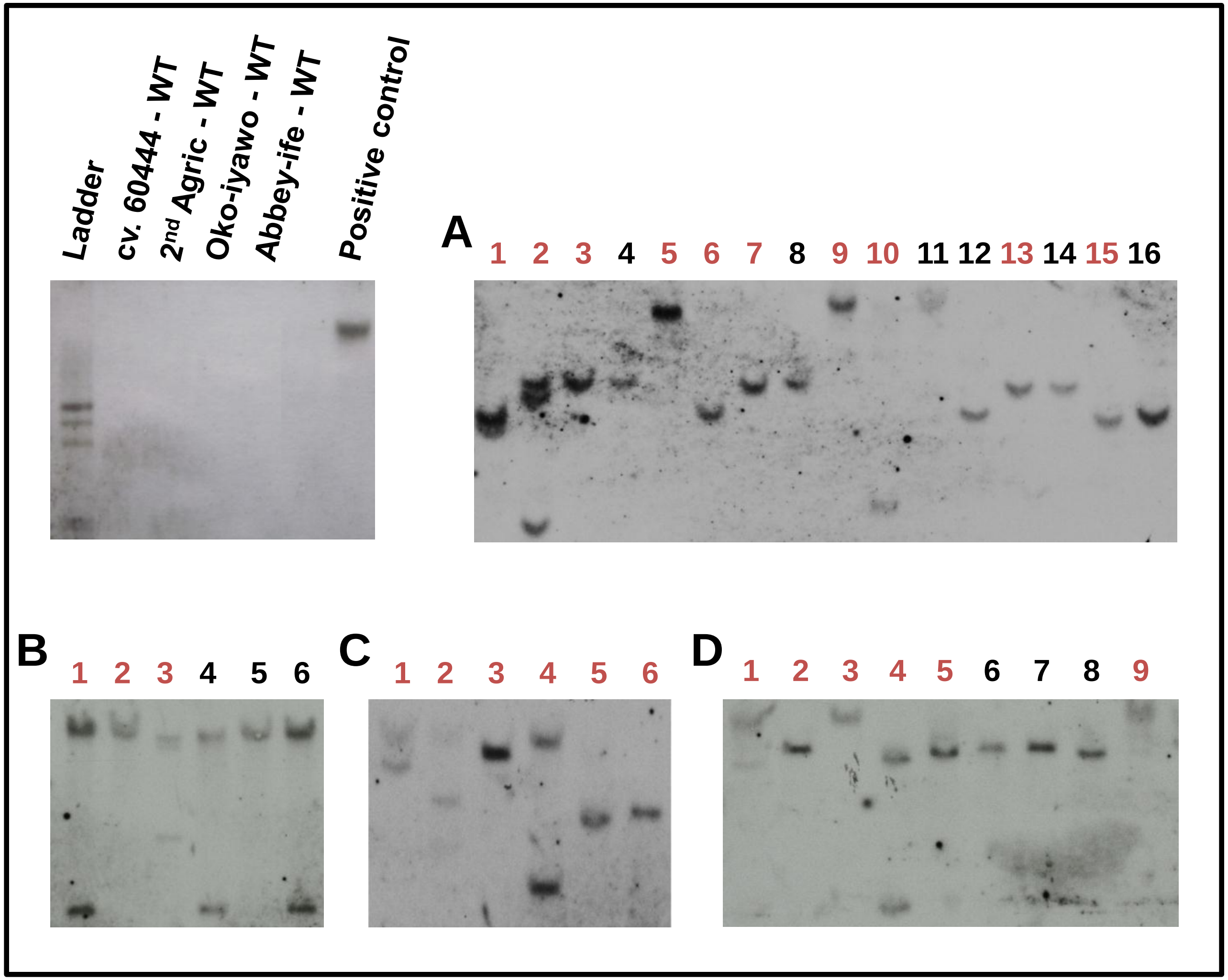

Supplementary Figure 1. Southern blot analysis of transgenic cassava plantlets. (A) 10 independent lines from 16 transgenic 2ndAgric plantlets generated with the DS procedure, (B) 3 independent lines from 6 transgenic Oko-iyawo plantlets generated with the DS procedure, (C) 6 independent lines from 6 transgenic Abbey-ife generated with the DS procedure, (D) 6 independent lines from 9 transgenic Oko-iyawo plantlets generated with the SS procedure. Independent lines are indicated by red labels. Southern blot has been repeated with *Pst*I restriction for lines with similar integration pattern to confirm independent integration event (data not shown).
